# Bibliometric and visual analysis of intraoperative hypotension from 2004 to 2022

**DOI:** 10.3389/fcvm.2023.1270694

**Published:** 2023-11-16

**Authors:** Jieyan Wang, Zile Liu, Yawen Bai, Guijie Tian, Yinghao Hong, Guo Chen, Yantong Wan, Hui Liang

**Affiliations:** ^1^ Department of Urology, People's Hospital of Longhua, Shenzhen, China; ^2^ College of Anesthesiology, Southern Medical University, Guangzhou, China; ^3^ School of Laboratory Medicine and Biotechnology, Southern Medical University, Guangzhou, China; ^4^ Guangdong Provincial Key Laboratory of Proteomics, Department of Pathophysiology, School of Basic Medical Sciences, Southern Medical University, Guangzhou, China; ^5^ Tendon and Injury Department, Sichuan Provincial Orthopedics Hospital, Sichuan, China

**Keywords:** intraoperative hypotension, VOSviewer, CiteSpace, visual analysis, bibliometric

## Abstract

**Background:**

Intraoperative hypotension (IOH) is a common complication occurring in surgical practice. This study aims to comprehensively review the collaboration and impact of countries, institutions, authors, journals, keywords, and critical papers on intraoperative hypotension from the perspective of bibliometric, and to evaluate the evolution of knowledge structure clustering and identify research hotspots and emerging topics.

**Methods:**

Articles and reviews related to IOH published from 2004 to 2022 were retrieved from the Web of Science Core Collection. Bibliometric analyses and visualization were conducted on Excel, CiteSpace, VOSviewer, and Bibliometrix (R-Tool of R-Studio).

**Results:**

A total of 1,784 articles and reviews were included from 2004 to 2022. The number of articles on IOH gradually increased in the past few years, and peaked in 2021. These publications were chiefly from 1,938 institutions in 40 countries, led by America and China in publications. Sessler Daniel I published the most papers and enjoyed the highest number of citations. Analysis of the journals with the most outputs showed that most journals concentrated on perioperative medicine and clinical anesthesiology. Delirium, acute kidney injury and vasoconstrictor agents are the current and developing research hotspots. The keywords “Acute kidney injury”, “postoperative complication”, “machine learning”, “risk factors” and “hemodynamic instability” may also become new trends and focuses of the near future research.

**Conclusion:**

This study uses bibliometrics and visualization methods to comprehensively review the research on intraoperative hypotension, which is helpful for scholars to better understand the dynamic evolution of IOH and provide directions for future research.

## 1. Introduction

More than 300 million people worldwide undergo surgery each year (1). How to reduce the incidence of postoperative complications has always been the focus and goal of medical workers. Intraoperative hypotension is a highly prevalent complication of non-cardiac surgery, and there is also a lot of evidence that IOH is strongly associated with the occurrence of other serious complications (2–4). There is still a lack of clear criteria for the definition and diagnosis of IOH, and the commonly used definition is that the patient has any episode of systolic blood pressure less than 80 mmHg or at least one episode of systolic blood pressure more than 20% below baseline (5). The pathophysiological mechanism of IOH is related to the self-regulation mechanism of human blood pressure and the patient's own factors such as age, gender, and surgical operation during surgery (6, 7). More in-depth studies remain to be conducted.

It is known from previous studies that the human cardiovascular system has the ability to regulate pressure within a certain range and this self-regulation ability varies significantly among different people (8, 9). The stability of arterial pressure ensures normal perfusion of all vital organs, when fluctuations in systemic blood pressure occur, certain vital organs, such as the brain and kidneys, can regulate their own blood flow within a certain range, thus minimizing the impact of fluctuations in overall blood pressure (6–9). However, once fluctuations in external blood pressure exceed the range of autonomic regulation of organs, serious adverse consequences, such as myocardial injury after noncardiac surgery (MINS) and acute kidney injury (AKI), can occur (4).

Meanwhile, with the rapid development of machine learning and other biomedical engineering fields, more and more algorithms have emerged to predict the occurrence of IOH (10). Few attempts have been made to systematically analyze the scientific results and the current status of the field from a global perspective. Therefore, there is an urgent need for a suitable visualization method to reveal the global status, future trends and hotspots of IOH.

The open science movement is considered the most critical change in bibliometrics since the emergence of the field in the 1960s. The free sharing of a wide range of scientific results on the Internet has transformed all aspects of bibliometric practice, including data collection, infrastructure, definitions, and metrics collection. Bibliometric methods can be used to explore the impact of research areas, researchers, and specific papers, or to mine the literature for impact or scholarly value in each research area (10–13).Bibliometric analysis has been applied to medical fields such as stroke (12), ERAS (13), and anesthesiology (14). However, there are still relatively few bibliometric studies on IOH. Therefore, this review systematically analyzes IOH research to assess the current status and hotspots in the field.

## 2. Materials and methods

### 2.1. Data sources

Our bibliometric analyses are based on the Web of Science Core Collection (WOSCC), the widely used dataset in bibliometrics today (15, 16), which has a set of data fields to multidimensionally support our analyses. We used “TS = intraoperative hypotension” as a search tool to retrieve literature and limited the document types to articles and reviews, resulting in 1,784 documents. All documents above are in English.

### 2.2. Data collection

All results were searched on WOSCC using the above formula and exported as plain text literature in formats txt and csv. The literature search was conducted on Oct 1, 2022 to prevent possible bias introduced by database updating.

### 2.3. Data analysis

Visual analysis was performed on Microsoft Excel 365, Bibliometrix (R-Tool of R-Studio) (17), VOSviewer (18), and CiteSpace (19). The advantages and disadvantages of the above econometric analysis software were also discussed in recent literature, and thus were not elaborated here (20). CiteSpace 6.1.R2 Advanced was used to visually analyze country distribution, institution distribution, subject area distribution, keyword timeline, references, keywords, and literature bursts from the above-extracted data. On VOSviewer 1.6.18, we visually analyzed country distribution, institution distribution, and author distribution from the extracted data. With Bibliometrix (R-Tool of R-Studio), we visualized country distribution, references, and keywords using R-Studio. Since all raw data used here were obtained from public databases, no ethical review was required.

## 3. Results

### 3.1. Trends and contributions of countries/regions to global publications

**Figure 1:**
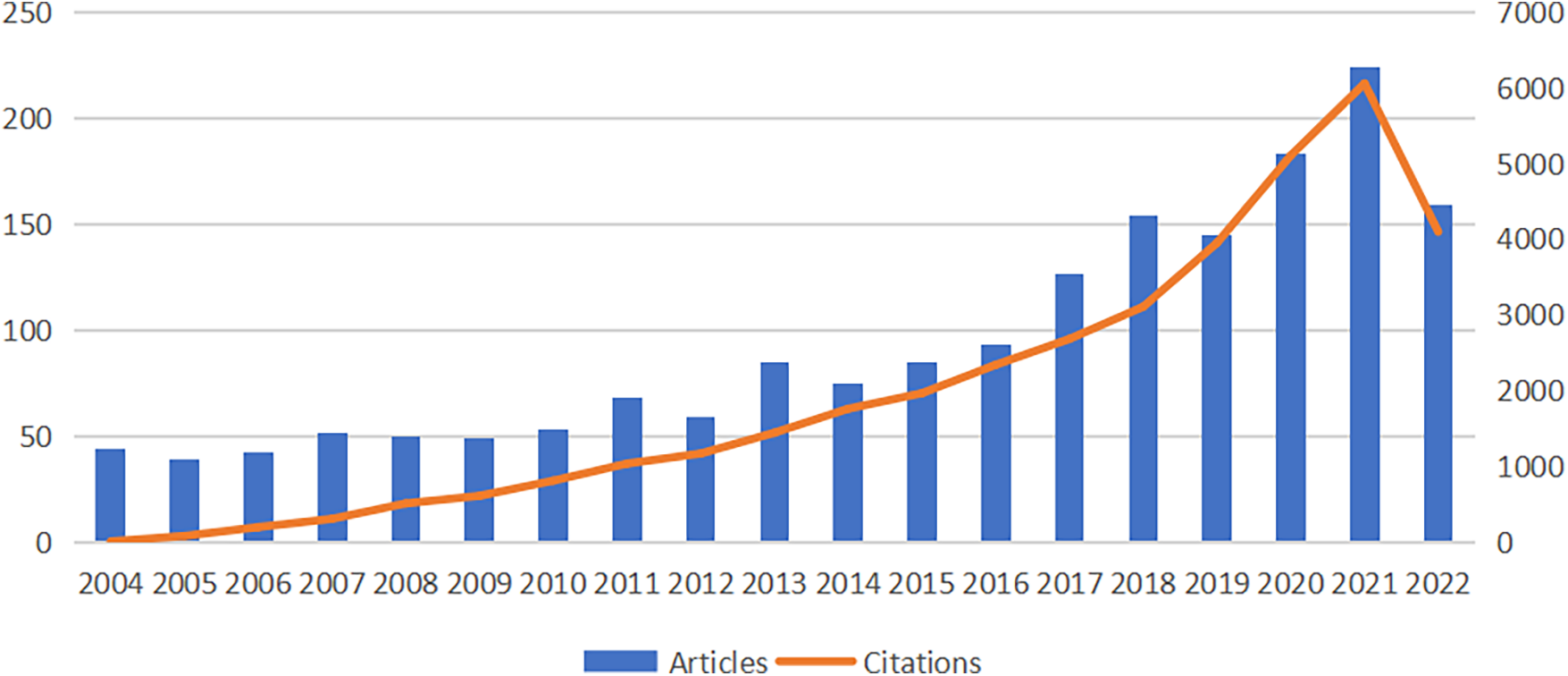
Trends in the publication and citation frequency of IOH-related literature (2004–2022).

In the present study, we collected 1,784 publications on intraoperative hypotension from the Web of Science core collection (WoSCC) published in 76 journals between 2004 and 2022. As shown in Figure 1, in the first 7 years (2004–2010), the number of articles published on IOH was basically stable at about 45 per year, with a small fluctuation. Since 2011, the research on IOH has generally shown an increasing trend and reached a peak in 2021 (*n* = 224). To get a conclusion, the above data suggested that in the last decade, IOH has been continuing growth in research interest which will evoke a broad research outlook.

**Figure 2:**
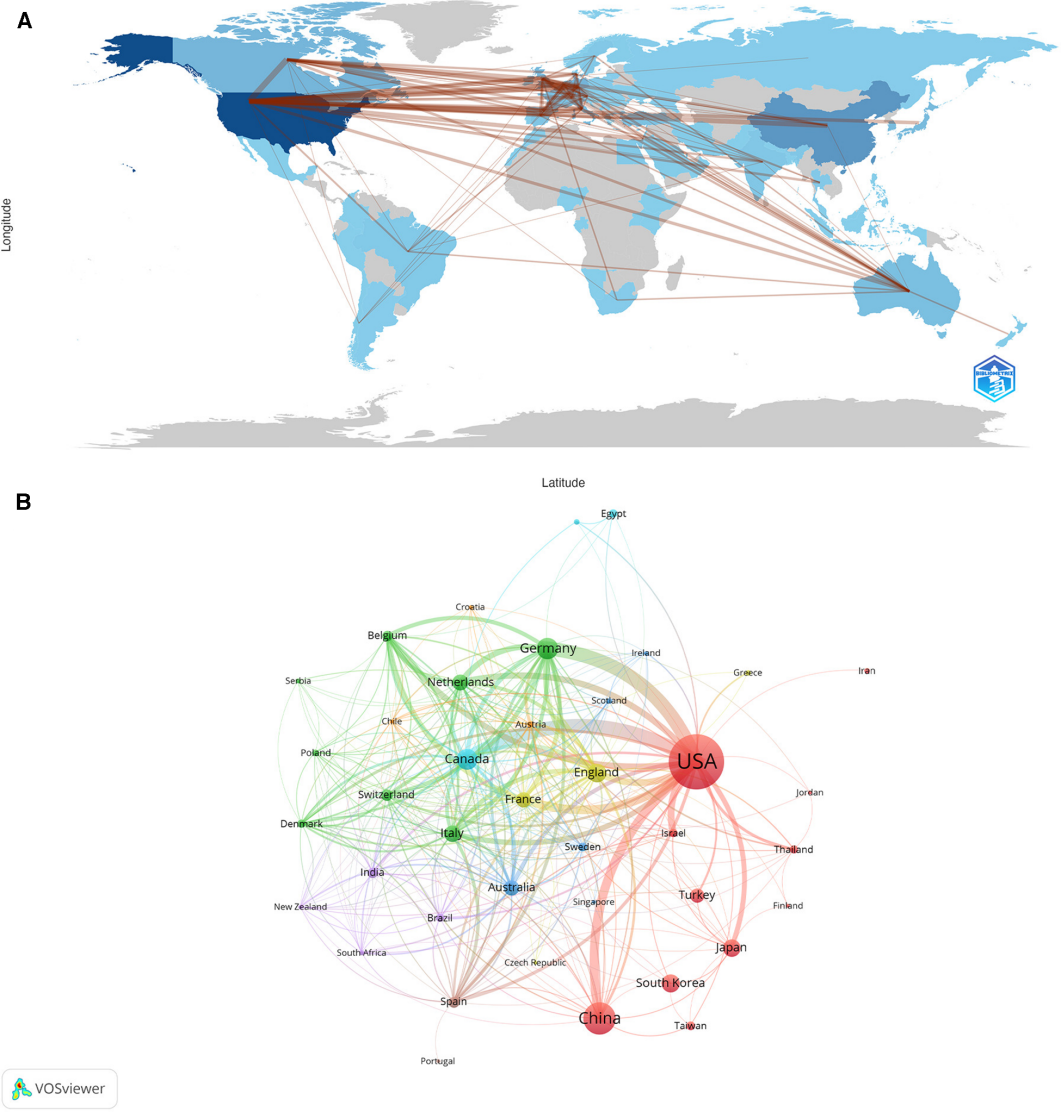
Visualization diagram of each country/region regarding IOH. (**A**) Countries/regions involved in IOH-related research. The links between countries/regions indicate their collaborations and connections. (**B**) Analysis of collaborative network of countries/regions. Different nodes represent different countries or regions. Different colors represent clusters with different affinities. Publications of countries was shown in the sizes of nodes.

From 2004 to 2022, a total of 75 countries/regions were involved in intraoperative hypotension related surveys between 2004 and 2022. The top 10 of countries/regions according to publications were shown in Table 1. Among them, authors from the United States published the highest number of articles on this topic (*n* = 659), followed by China (*n* = 249) and Germany (*n* = 124). Unsurprisingly, America occupied the highest number of citations (18,987), followed by Canada (5,068) and England (2,857) ranked third. The average number of citations per article published by Canada was 44.07, followed the Netherlands (37.54) and England (30.39). Despite ranking second in the number of publications, China has only 2,502 citations, the second lowest average number of citations per article among the top 10 productive countries. Moreover, in Figure 2A, it is noteworthy that the links between countries and regions are mainly concentrated between North America and Europe, and between North America and East Asia. And the countries with more publications also cooperate more closely with each other than those with fewer publications. Figure 2B shows the international cooperation by publication volume. The results are divided into several clusters according to their closeness of cooperation, which are represented in different colours. Each node represents a country/region, and the radius of the node increases with its contribution to IOH-related research. The connections between nodes indicate the collaborative relationship between individual countries and regions, and the thickness of the links is positively correlated with the depth of collaboration. The United States also had the strongest total link strength in this field and close cooperation with several countries such as China, Germany and Canada. Notably, Canada, the Netherlands, and England were in the leading position according to their average citation of per article, demonstrating the researches from those countries were influential. The contributions of publications in this field were uneven in terms of countries.

**Table 1:** Top 10 country/region in terms of the number of publications, frequency of citations, and total association intensity.

Rank	Countries	Documents	Citations	Average citations	Total link strength
1	USA	659	18,987	28.81	290
2	China	249	2,502	10.05	68
3	Germany	124	2,448	19.74	172
4	Canada	115	5,068	44.07	140
5	England	94	2,857	30.39	140
6	South Korea	89	779	8.75	9
7	Japan	87	965	11.09	32
8	Italy	78	1,951	25.01	113
9	Netherlands	72	2,703	37.54	102
10	Australia	68	1,380	20.29	88

### 3.2. Contributions of institutions

Between 2004 and 2022, a total of 1,938 institutions were involved in intraoperative hypotension research. In Table 2, we showed the top 10 institutions in publications, citations, and total link strength. Among the top 10 institutions in publications, except for the University of Toronto, ranked 4th, from Canada, the 5th one Capital Medical University from China, and the 10th ranked Outcomes Research Consortium is an international organization, the remaining seven institutions were all from the United States. Cleveland Clinic was regarded as the relatively productive and influential institution in the field of IOH-related research with 69 publications, followed by Mayo Clinic (27). Simultaneously, Cleveland Clinic was cited 2,446 times, which was much higher in the number of citations than that of any other institutions. The second most-cited institution was McMaster University with 1,580 times. Unexpectedly, China, the country with the second largest number of publications, had only one Chinese university Capital Medical University on the list of publishing volumes, and was absent from the other two rankings, implying its low centralization of studies in this field.

**Table 2:** Top 10 institutions in terms of volume of publications, citations, and total link strength.

Rank	Institution	Publications	Rank	Institution	Citations	Rank	Institution	Total link strength
1	Cleveland Clin	69	1	Cleveland Clin	2,446	1	Cleveland Clin	70
2	Mayo Clin	30	2	Mcmaster Univ	1,580	2	Outcomes Res Consortium	44
3	Duke Univ	29	3	Duke Univ	1,361	3	Univ Calif Irvine	35
4	Univ Toronto	28	4	Univ Michigan	1,338	4	Univ Calif Los Angeles	30
5	Capital Med Univ	26	5	Univ Pittsburgh	1,164	5	Univ Libre Bruxelles	29
6	Harvard Med Sch	25	6	Univ Toronto	1,129	6	Univ Paris Saclay	28
7	Univ Calif Los Angeles	24	7	Mayo Clin	875	7	Univ Calif San Diego	26
8	Univ Pittsburgh	24	8	Univ Med Ctr Utrecht	874	8	Univ Med Ctr Hamburg Eppendorf	24
9	Ohio State Univ	23	9	Univ Calif San Francisco	823	9	Univ Melbourne	22
10	Outcomes Res Consortium	23	10	Inserm	747	10	Mayo Clin	20

**Figure 3:**
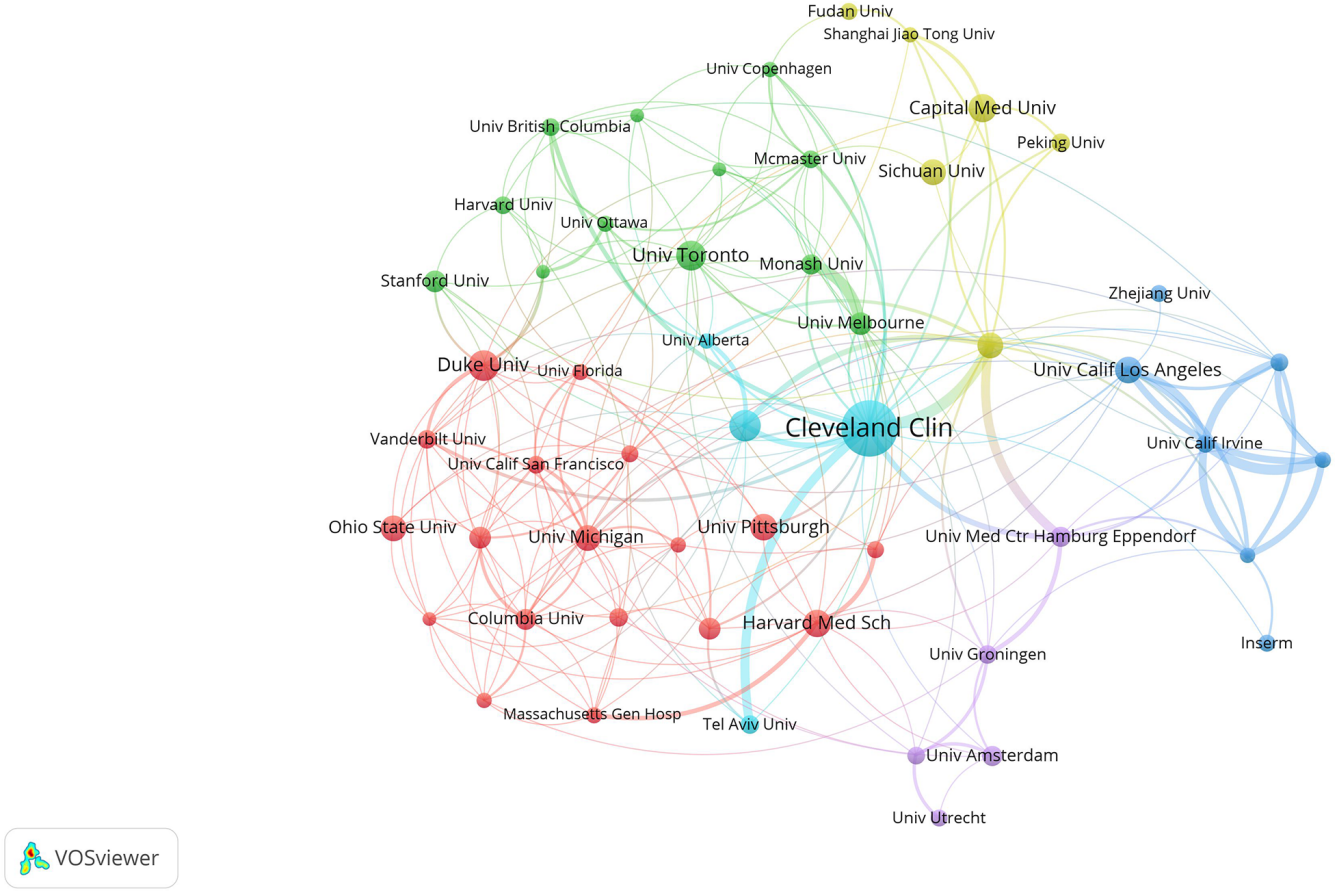
Visualization diagram of each institution regarding IOH. Applying VOSviewer, the collaborative network of institutions. Different nodes represent different institutions. Different colors represent clusters with different affinities. Node size represents the publication volume of the institution.

In order to analyze the cooperation between different institutions, VOSviewer was applied to visualize the relationship map (Figure 3). In the figure, nodes of different colors represented different clusters based on co-occurrence intimacy, the size of the nodes indicated the frequency of occurrence, and the links between nodes indicated the co-occurrence relationship. As is shown, those institutions were clarified into six clusters, implying a strong regional identity. It is clear that in the complex network, the yellow cluster includes universities in China, Univ Calif Los Angeles and Univ Calif Irvine in the United States are the main ones in the blue cluster and the purple cluster are mainly Dutch universities. These clusters lack close connections with each other and are relatively isolated from the center. Thus, the cyan and red clusters mainly consisting of universities and institutions in the United States had the strongest intermediary centrality. The Cleveland Clinic, in particular, has also played an important role in international collaboration with its prolific academic output. The above data suggest that most of the cooperation in this field of research was limited to the domestic level, and international cooperation should be strengthened among all countries, and strong cooperation is needed. Moreover, it was for China and some EU countries that should promote collaboration with other international institutions.

### 3.3. Contributions of authors and co-authors

**Figure 4:**
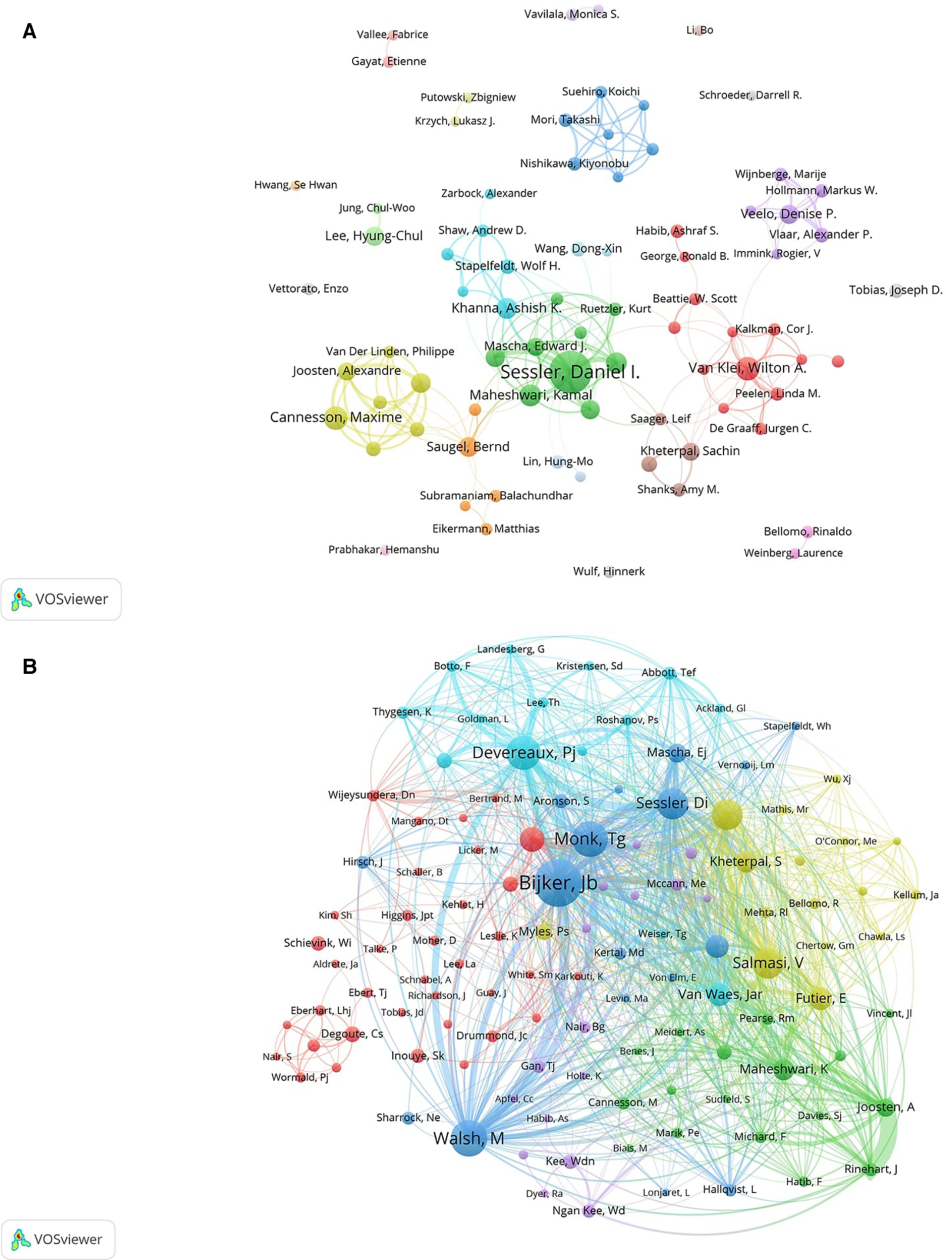
Visualization of association graphs of co-authors and co-cited authors in IOH. (**A**) Visualization of the network of collaboration between authors. The size of the node represents the publication of the author. Identical colors represent internal connections within clusters. The thickness of the line between the authors represents the closeness of the cooperative relationship. (**B**) Network of co-cited authors. Sizes of nodes represent the association intensity of co-cited authors.

A total of 8,999 authors were involved in the intraoperative hypotension(IOH) studies between 2004 and 2022. According to Prices law, authors with 5 or more publications in the field of IOH were core authors. Therefore, the top 10 authors were listed in Table 3. Sessler Daniel I from McMaster University with the possession of a total of 43 publications and 2,789 citations was credited as the influential and productive author in this field. It is worth noting that although Kurz Andrea is only tied for 9th in the ranking of publications, his article is ranked second in the number of citations, which shows his high academic achievements and influence. The analysis of the co-authorship figures (Figures 4A,B), offered us information about the iconic or representative scholars and core research strengths of the field of IOH. A total of 1,784 publications from 8,999 authors were calculated, and we applied VOSviewer to visualize the co-authorship network. As shown in Figure 4A, 22 clusters were divided according to the closeness of the collaborative relationship between the co-authors. Firstly, the biggest node is centered on Sessler Daniel I, and someone who had the closest connection with him were two authors, Maheshwari Kamal, and Mascha Edward J. Two of them also enjoyed a high academic reputation in this filed, suggesting that collaboration between outstanding scholars contributes to the development of the OLV field. Notably, the dark blue group(Mori Takashi, Suehiro Koichi and Nishikawa Kiyonobu), the cyan group(Putowski Zbigniew and Krzych Lukasz J), the light green group(Jung Chul-Woo and Lee Hyung-Chul), the light red group(Vallee Fabrice and Gayat Etienne), and the purple group(Bellomo Rinaldo and Weinberg Laurence) were not connected with any other groups. In summary, scholars in the field of IOH are mostly limited to intra-institutional or intra-national collaborations. The lack of cooperation with other institutions and international cooperation will limit the advancement of OLV technology and the innovation of thoracic anesthesia management methods.

**Table 3:** Top 10 authors in terms of publications, citations, and co-citations.

Rank	Author	Documents	Rank	Author	Citations	Rank	Author	Co-citations
1	Sessler, Daniel I	43	1	Sessler, Daniel I	2,789	1	Bijker, Jb	434
2	Cannesson, Maxime	17	2	Kurz, Andrea	1,689	2	Walsh, M	287
3	Van Klei, Wilton A	17	3	Van Klei, Wilton A	1,418	3	Monk, Tg	280
4	Maheshwari, Kamal	15	4	Turan, Alparslan	1,023	4	Devereaux, Pj	253
5	Khanna, Ashish K	14	5	Bijker, Jilles B	872	5	Sessler, Daniel I	225
6	Turan, Alparslan	14	6	Kalkman, Cor J	848	6	Sun, Ly	219
7	Rinehart, Joseph	13	7	Yang, Dongsheng	814	7	Salmasi, V	217
8	Saugel, Bernd	13	8	Van Wolfswinkel, Leo	773	8	Reich, Dl	150
9	Kurz, Andrea	12	9	Mascha, Edward J	741	9	Wesselink, Em	136
10	Lee, Hyung-Chul	12	10	Maheshwari, Kamal	733	10	Futier, E	135

Next, we analyzed the co-cited author relationship, which partially reflected the same peculiarities among the co-cited authors in this field (Figure 4B). Authors were divided into six clusters and the size of the nodes indicated the frequency of their occurrence as co-cited authors. The network map of the co-cited-authors reflected their research direction and academic cross-development in the academic community. In the blue cluster, Monk Tg, Bijker Jb, Sessler Di and Walsh M had relatively large nodes, which means that they had higher co-citation strength than other authors. Only a small part of the red group(Eberhart Lhj, Degoute Cs, Nair S, and Wormald Pj) was sparsely connected to other groups, It's worth noting that a node of the red group(Schievink Wi) was isolated from any other groups. To get a conclusion, the directions of research on IOH were generally concentrated, yet the focus was varied.

### 3.4. Analysis of journals

Between 2004 and 2022, a total of 501 journals published articles and reviews on the topic of intraoperative hypotension. The top 10 most productive journals published 504 papers, accounting for 28.25% of all 1,784 publications (Table 4). In terms of publication volume, citation frequency and co-citation intensity, *Anesthesiology*, *Anesthesia* and *Analgesia* and *British Journal* of *Anaesthesia* all ranked in the top 3, indicating that these three journals have high academic status and authority in the field of IOH. According to JCR 2020 standard, half of the top 10 productive journals were elected as Q1 or Q2, demonstrating that they published high-quality and influential papers in the related field. Notably, although *Annals* of *Thoracic Surgery* and *Annals* of *Surgery* didn't occupy a place in the top 10 ranking of publications but ranked 4th and 8th relatively in the ranking of citations, implying that the publications of two journals gained a high level of scholarly authority in IOH research.

**Table 4:** Top 10 journals in terms of publication and citation, and top 10 cited journals.

Rank	Journal	Publications	IF(JCR2022)	JCR quatile
1	Anesthesia and Analgesia	109	6.627	Q2
2	Anesthesiology	64	8.986	Q1
3	British Journal of Anaesthesia	58	11.719	Q1
4	BMC Anesthesiology	49	2.376	Q2
5	Journal of Clinical Anesthesia	47	9.375	Q1
6	Journal of Cardiothoracic and Vascular Anesthesia	44	2.894	Q3
7	Pediatric Anesthesia	37	2.129	Q4
8	Journal of Anesthesia	36	2.931	Q3
9	Journal of Clinical Monitoring and Computing	31	1.977	Q4
10	Current Opinion in Anesthesiology	29	2.733	Q3
Rank	Journal	Citations	IF (JCR2022)	JCR quatile
1	Anesthesiology	6,023	8.986	Q1
2	Anesthesia and Analgesia	3,171	6.627	Q2
3	British Journal of Anaesthesia	2,210	11.719	Q1
4	Annals of Thoracic Surgery	883	5.102	Q2
5	Pediatric Anesthesia	768	2.129	Q4
6	Journal of Clinical Anesthesia	646	9.375	Q1
7	Journal of Vascular Surgery	607	4.86	Q2
8	Annals of Surgery	607	13.787	Q1
9	Journal of Bone and Joint Surgery-American Volume	592	6.558	Q1
10	Journal of Neurosurgery	484	5.408	Q1

**Figure 5:**
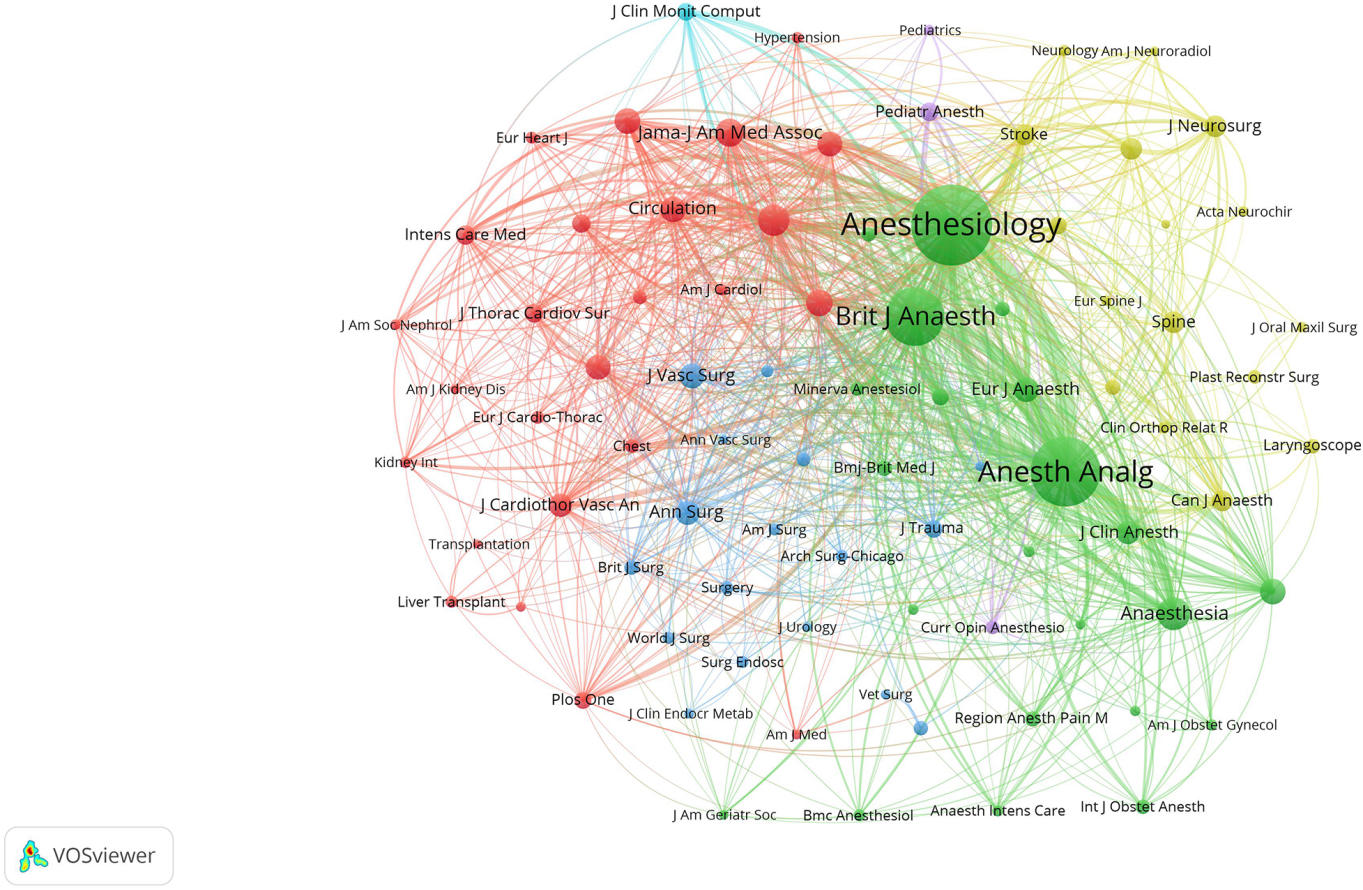
The node size represented the number of papers published, and the line represented the cooperation. Different colors each represent a closer cooperative relationship.

From Figure 5, it is shown that the co-citation network of journals consists of 6 clusters, showing the interactions and connections between journals, with the thicker line reflecting a closer relationship. The journals in the green cluster, most are anesthesiology category publications, among which *Anesthesiology* and *Anesthesia* and *Analgesia* were tend to be co-cited with *British Journal* of *Anaesthesia*, and all these were representative journals about anesthesiology. Journals focusing primarily on the causes and physiological processes of intraoperative hypotension were clustered in yellow. And the purple cluster focuses on the pediatric anesthesia category. The dark blue cluster and the yellow cluster focus primarily on studies at the surgical as well as neurosurgical level, and the main purpose of citing these journals is to review the available case studies to provide existing theoretical and empirical support for the development of the survey. The light blue cluster focuses primarily on the intraoperative surveillance category, and the purpose of citing these journals is primarily to provide technical-level support for the research. In general, journals in each cluster have their own similar research topics or specific internal logical correlation, representing various popular research directions of IOH.

### 3.5. Analysis of citations

The purpose of co-citation analysis was to master the papers that were frequently used in this research field and the journals that published these papers, therefore we formed the co-citation map of journals through VOSviewer.

Furthermore, we analyzed the top 15 cited literatures in this field between 2004 and 2022. As shown in Table 5, more than half of the articles are from *Anesthesiology.* The top one paper was *Relationship between Intraoperative Mean Arterial Pressure and Clinical Outcomes after Noncardiac Surgery: Toward an Empirical Definition of Hypotension*, and this retrospective analysis showing that an intraoperative mean arterial pressure (MAP) less than 55 mmHg were associated with adverse cardiac- and renal-related outcomes (4).The total citations and average annual citations of this paper were up to 756 and 75.6 respectively. Moreover, the second most cited paper was a prospective observational study, *Anesthetic management and one-year mortality after noncardiac surgery*, showing that intraoperative hypotension was also an independent predictor of increased mortality in the first postoperative year (relative risk = 1.036/min; *P* = 0.0125) (21). The ranked 3rd article was *Wound infection after elective colorectal resection*. This study indicated that a relatively higher BMI and IOH were identified as two independent risk factors for surgical site infection (SSI). However, the study was unable to elucidate the precise pathophysiological relationship between SSI and IOH, but hypothesized that poor tissue perfusion may contribute to SSI.

**Table 5:** Top 15 publications in terms of the frequency of citation.

Rank	Author	Article title	Journal	Cited	Year	Document type
1	Sessler DI, et al.	Relationship between intraoperative mean arterial pressure and clinical outcomes after noncardiac surgery: toward an empirical definition of hypotension	Anesthesiology	756	2013	Article
2	Monk TG, et al.	Anesthetic management and one-year mortality after noncardiac surgery	Anesthesia and Analgesia	595	2005	Article
3	Smith RL, et al.	Wound infection after elective colorectal resection	Annals of Surgery	425	2004	Article; Proceedings Paper
4	Sessler DI, et al.	Relationship between intraoperative hypotension, defined by either reduction from baseline or absolute thresholds, and acute kidney and myocardial injury after noncardiac surgery a retrospective cohort analysis	Anesthesiology	412	2017	Article
5	Hemmes SNT, et al.	High vs. low positive end-expiratory pressure during general anaesthesia for open abdominal surgery (PROVHILO trial): a multicentre randomised controlled trial	Lancet	382	2014	Article
6	Bijker JB, et al.	Incidence of intraoperative hypotension as a function of the chosen definition—literature definitions applied to a retrospective cohort using automated data collection	Anesthesiology	360	2007	Article; Proceedings Paper
7	Sun LY, et al.	Association of intraoperative hypotension with acute kidney injury after elective noncardiac surgery	Anesthesiology	355	2015	Article
8	Lienhart A, et al.	Survey of anesthesia-related mortality in France	Anesthesiology	345	2006	Article
9	Futier E, et al.	Effect of individualized vs standard blood pressure management strategies on postoperative organ dysfunction among high-risk patients undergoing major surgery a randomized clinical trial	JAMA-Journal of the American Medical Association	330	2017	Article
10	Blaudszun G, et al.	Effect of perioperative systemic alpha 2 agonists on postoperative morphine consumption and pain intensity systematic review and meta-analysis of randomized controlled trials	Anesthesiology	300	2012	Article
11	Sessler DI, et al.	Hospital stay and mortality are increased in patients having a triple low of low blood pressure, low bispectral index, and low minimum alveolar concentration of volatile anesthesia	Anesthesiology	283	2012	Article
12	Monk TG, et al.	Association between intraoperative hypotension and hypertension and 30-day postoperative mortality in noncardiac surgery	Anesthesiology	275	2015	Article
13	Gooch MR, et al.	Complications of cranioplasty following decompressive craniectomy: analysis of 62 cases	Neurosurgical Focus	272	2009	Article
14	Conrad MF, et al.	Thoracoabdominal aneurysm repair: A 20-year perspective	Annals of Thoracic Surgery	267	2007	Article; Proceedings Paper
15	Donaldson AJ, et al.	Bone cement implantation syndrome	British Journal of Anaesthesia	267	2009	Article

**Table 6:** Top 20 keywords with the most frequent occurrences.

Rank	Keyword	Occurrences	Total link strength	Rank	Keyword	Occurrences	Total link strength
1	Hypotension	188	304	11	Haemodynamics	42	73
2	Anesthesia	138	207	12	Intraoperative	42	94
3	Dexmedetomidine	75	107	13	Blood loss	39	41
4	Intraoperative hypotension	64	76	14	Spinal anesthesia	36	57
5	Acute kidney injury	63	86	15	Propofol	35	54
6	Surgery	60	93	16	Mortality	29	49
7	General anesthesia	58	64	17	Postoperative complications	29	43
8	Blood pressure	57	104	18	Elderly patients	28	50
9	Risk factors	46	66	19	Outcome	28	49
10	Complications	44	55	20	Cesarean section	27	42

**Figure 6:**
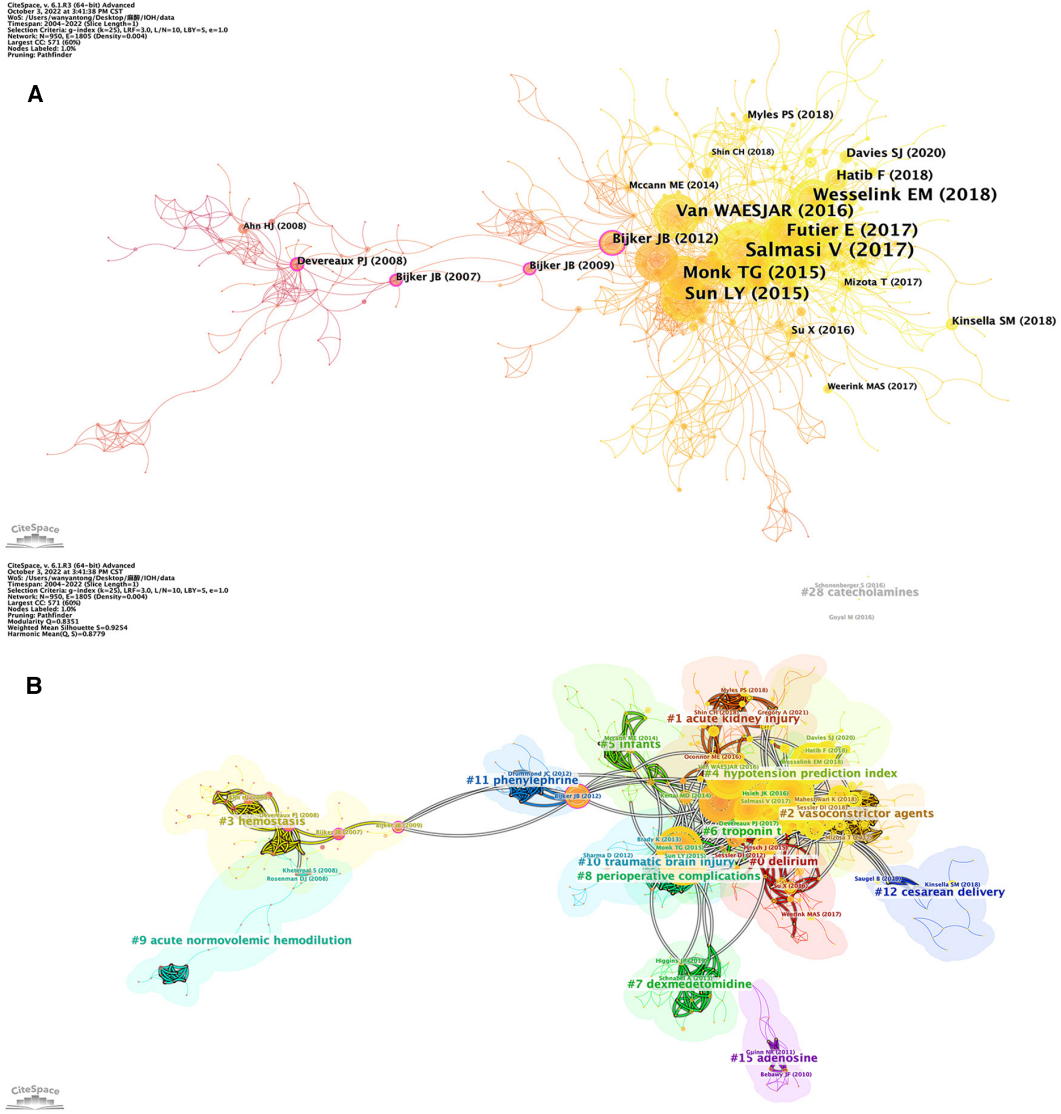
(**A**) Application of citespace, literature relation network diagram. The different colors of the nodes indicate additional years, from purple to yellow, the later. The node size indicates the frequency of the reference. (**B**) The heat topic view of keywords. The smaller the number is, the larger the cluster, with #0 being the largest cluster. Nodes size reflects co-citation frequency, and the links between nodes indicate co-citation relationships.

Next, the relationship among the studies in temporal dimension was analyzed on CiteSpace, demonstrating the frequency of papers of co-citations in a specific time (Figure 6A). Each node represented an article, and its size symbolized the frequency of being cited in the IOH field. The color of the node indicated its year, from red to yellow, the later the time.The number of top papers cited is related to how often research was published that year. Among all hot topics, the proportion of highly cited articles is relatively high. Papers with high citations are more likely to be known as hot papers, which confirms the correlation between research popularity and citations to some extent. *Incidence of intraoperative hypotension as a function of the chosen definition—Literature definitions applied to a retrospective cohort using automated data collection* (5), *Intraoperative Hypotension and 1-Year Mortality after Noncardiac Surgery* (22)*,* and *Intraoperative Hypotension and Perioperative Ischemic Stroke after General Surgery A Nested Case-control Study* by Bijker JB et al. were marked with purple rings in high betweenness centrality(≥0.10) which is usually leveraged to probe the important turning points that may result in transformative discoveries and serve as a bridge.

The clustering is divided into 28 categories based on the degree of correlation between literatures (Figure 6B). The size of each node was proportional to the co-citation frequency of the article (23). The category with the largest number of published articles is #0 delirium. The cluster with the most cited papers was #4, with the keyword as known as hypotension prediction index. By comparison, the clusters like #9 acute normovolemic hemodilution, #3 hemostasis, and #28 catecholamines were almost at the edge of the figure. The clusters #9 acute normovolemic hemodilution and #3 hemostasis, with relatively more citations, perhaps were the last research hotspots and now is no longer scenery due to the rapid development of health care and shortage of the leading figure in this topic. The cluster #28 catecholamines were small in scale as well because studies on the effect of catecholamines in perioperative had gradually increased yet it was not strongly associated with IOH. Furthermore, it could be indicated that roughly the clusters could be separated into 2 parts based on time, a pre-2013 category and a post-2013 category, consistent with the transition in 2013 shown in Table 1, demonstrating the hotspots in IOH were switched.

**Figure 7:**
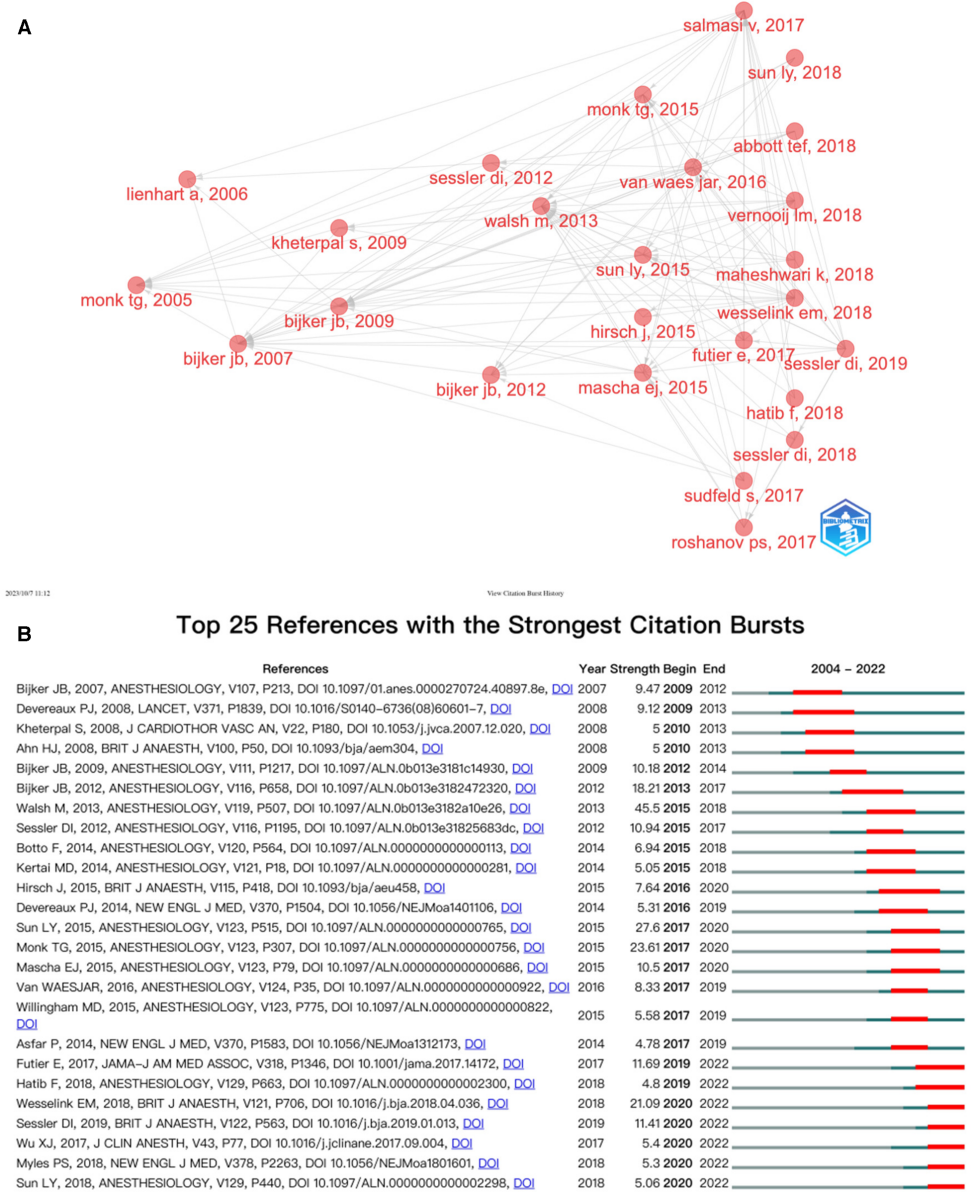
(**A**) Association between the top 20 citation bursts. (**B**) The top 25 references with the strongest citation bursts.

Briefly, a more concise analysis of high cited references shows clearly the structure of development is displayed in Figure 7A. The earliest high cited article is “*Anesthetic management and one-year mortality after noncardiac surgery*”, Monk TG,et al. published in 2005, suggesting that intraoperative anesthetic management may affect outcomes over longer time periods than previously appreciated. Subsequently, 2015(4 articles) and 2018(7 articles) had the most highly cited articles, and these articles served as a link between the previous phase and the next phase. Figure 7B shows the top 25 references with the strongest citation bursts. The first two bursts occurred in 2009. They are titled “*Incidence of Intraoperative Hypotension as a Function of the Chosen Definition: Literature Definitions Applied to a Retrospective Cohort Using Automated Data Collection*”by Bijker JB, et al. and “*Effects of extended-release metoprolol succinate in patients undergoing non-cardiac surgery (POISE trial): a randomised controlled trial*”by Devereaux PJ, et al. It is worth noting that the paper “*Relationship between Intraoperative Mean Arterial Pressure and Clinical Outcomes after Noncardiac Surgery: Toward an Empirical Definition of Hypotension*” by Walsh M, et al. published in 2013 is with the strongest burst (Strength = 45.5), and its burst duration occurred from 2013 to 2018, which is a key paper to fill the gap in the definition of IOH and lay the foundation for the further research on the prediction and prevention of IOH. There are 7 citation bursts until 2022, indicating that the IOH-related research is ongoing.

### 3.6. Analysis of keywords

**Figure 8:**
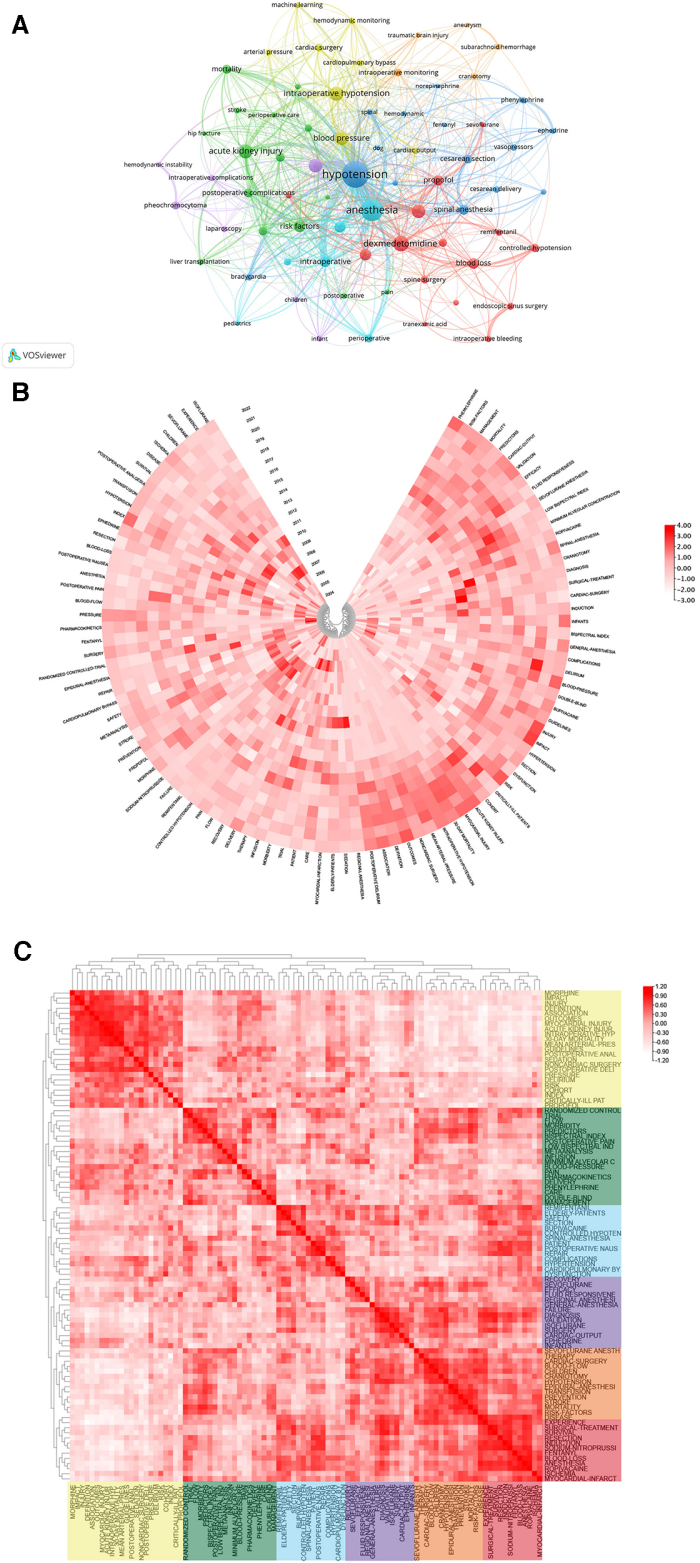
(**A**) Keyword co-occurrence network relationship diagram. hypotension has most co-occurrence with others keywords. Heatmap analysis of IOH-related keywords. (**B**) Annual heatmap from 2004 to 2022. The annual heat value of each keyword is obtained by dividing the number of citations in that year by the total number of citations in that year. (**C**) Keyword relevance heatmap. Keywords with high popularity in similar time periods are clustered into one category and marked with different colors.

Keywords condensed the core and essence of a paper, and keyword co-occurrence analysis can reveal research hot spots in a specific field. In Table 6, we analysed the top 20 keywords with the most frequent occurences. It showed that “Hypotension” and “Anesthesia” enjoyed the two highest occurences, relatively in 188 and 138 times. As shown in Figure 8A, the result showed a total of 71 keywords (>10 times) in the 1,784 papers, identified and classified into seven clusters by VOSviewer. In the blue cluster, the keyword “hypotension” was the most frequent keyword(188 times), which is consistent with our research theme, and the following keywords were frequently mentioned in linical anesthesia, such as “spinal anesthesia” (36 times), “cesarean section” (27 times) and “bradycardia” (18 times). Moreover, the blue cluster centered as “hypotension” turned out to be centralized in the diagram and have frequent connections with any other keywords, revealing that they served as a solid theoretical background for other research. The green cluster is dominated by keywords “acute kidney injury”, “postoperative complications” and “mortality” for other complications associated with IOH. The grey cluster inclued “anethesia"(138 times), “haemodynamics” (42 times), “intraoperative” (42 times), and “perioperative"(26 times), relating to perioperative medicine. In the red cluster, there were “dexmedetomidine"(75 times), “general anesthesia” (58 times), “complications” (44 times), and “blood loss” (39 times). The key words of purple cluster are related to “hemodynamic instability”, such as “pheochromocytoma”, “children” and “laparoscopy”. The orange cluster had keywords “intraoperative monitoring”, “craniotomy”, “traumatic brain injury” and “subarachnoid hemorrhage”, which were related to cranial surgery and complications. The cluster was on the edge of the network and was small in scale, indicating that this area of research had received little attention.

As was presented in Figure 8B, it shows the keywords' annual popularity (number of citations in the year/total citations in the year) from 2004 to 2022. Keywords such as isoflurane and sodium-nitroprusside have had relatively low annual popularity in recent years. In contrast, the annual popularity of keywords about the complications of IOH such as infants, injury, acute kidney injury, and risk have been relatively high in recent years, suggesting that these keywords represent emerging frontier areas. Figure 8C shows the popularity correlation of keywords, where keywords with high popularity in similar periods are clustered into different clusters marked with different colors. The results show that there are six clusters: the yellow cluster (morphine, injury, delirium, etc.), green cluster (randomized control, flow, morbidity, etc.), blue cluster (remifentanil, elderly-patients, complications, etc.), purple cluster (cardiac-output, ephedrine, cardiac-surgery, etc.), orange cluster (sevoflurane anesth, therapy, children, etc.),and red cluster (experience, surgical-treatment, resection, etc.). This indicates that keywords within the same cluster have higher popularity in the same period.

**Figure 9:**
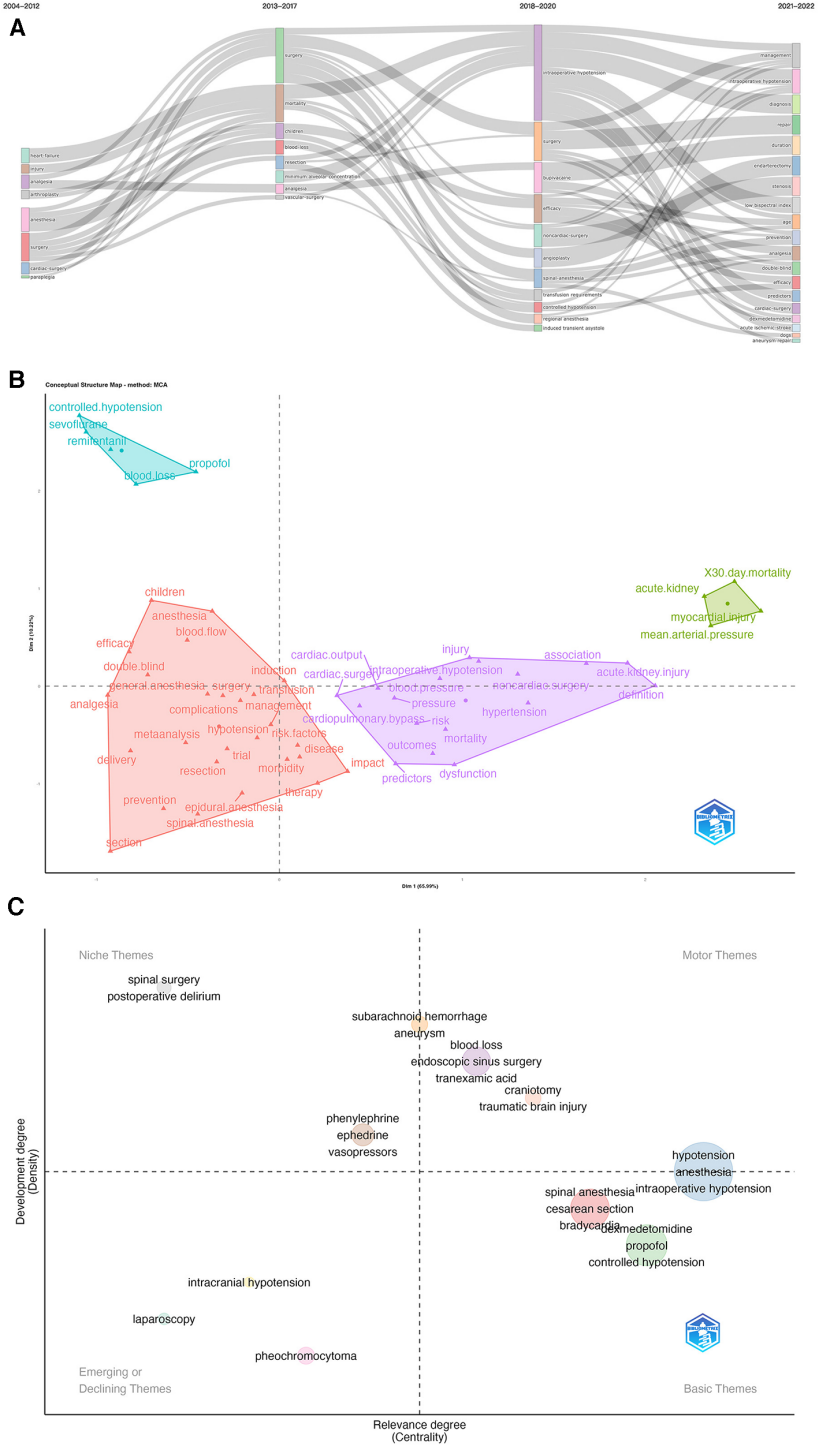
(**A**) Thematic evolution cutting in 4 phases. Keywords are ranged according lengths of keywords representing occurences. (**B**) Conceptual structure map using method MCA. Different colors mean different clusters. (**C**) Thematic map. Different colors mean different clusters, and X axis shows relevance degree as well as Y axis shows development degree.

According Bibliometrics, to all the keywords, as the most influential keywords, it were principal component analysis, analgesia, and surgery from 2004 to 2012; surgery and mortality from 2013 to 2017; intraoperative hypotension, surgery and bupivacaine from 2018 to 2020; and management and intraoperative hypotension from 2021 to 2022 (Figure 9A). Moreover, we analyzed the word map and thematic map in the field of IOH. In Figure 9B, it displays the focus of IOH's research attention are on 4 categories and how each category is described by the keywords individually. After understanding the general classification of research topics, we can understand the future research direction from Figure 9C. In Figure 9C, the horizontal axis represents the centrality and the vertical axis represents the density, according to which 4 quadrants are plotted, the first quadrant (upper right corner): motor themes, which represent topics that are both important and have developed well, such as traumatic brain injury and craniotomy; Second quadrant (upper left): niche themes, indicating that the topic has developed well but is not important to the current field, spinal surgery and postoperative delirium in the IOH field belong to this quadrant; Third quadrant (bottom left): emerging or declining themes, which represent marginal themes, which may not be well developed or may just emerge or are about to disappear, such as laparoscopy and pheochromocytoma; Quadrant 4 (bottom right): basic themes, which are topics that are important to the field but have not been well developed, are generally basic concepts, such as controlled hypotension and propofol.

## 4. Discussion

### 4.1. General distribution

Definitely, the index of citations and publications can serve as a necessary indicator for the development of a certain field. In the past 19 years, research in related fields has generally shown an upward trend, especially after 2013. Based on the statistical data of publications and citations and the analysis of hotspots, we firmly believe that the IOH field will still be prosperous and have more branches on it due to the development of perioperative medicine.

The United States has made the most significant contribution to the field of IOH, accounting for 1/3 of the global total publications (29.58%) or total citation frequency (36.25%). The United States’ leading position in this field was mainly attributed to its professional researchers, advanced equipment, first-class institutions and universities, and influential journals. Notably, this was despite China ranking second in the total number of publications but citations did not correspond to output, with an average citation rate less than half the global average and significantly lower than other high-producing countries. It shows that the Chinese research in the field of intraoperative hypotension lacks the influence recognized and evaluated by the world.

A similar spectrum also can apply to the distribution of institutions, with only one Chinese institution entering the top 10 productive institutions and without occupying a place in the ranking of citations. Capital Medical University is the most prolific institution in China but just ranked 36th in the view of the world. Combined with the above analysis, IOH research in China is in a relatively low concentration and has not formed a system and scale. Therefore, institutions and universities in China should concentrate more resources to support related research. The distribution of institutions confirms the dominance of America in this field. Cleveland Clinic and Mayo Clinic are two renowned medical institutions, where millions of patients visit every year. With the most cutting-edge medical technology, they have multi-dimensional clinical data on patients that can be used to conduct various types of research, and naturally, their publication volume is large. Furthermore, institutions like universities, driven by their own academic excellence, are encouraged to cooperate with the above two renowned medical laboratories with their massive clinical databases to promote the conversion of basic theoretical research into clinical trials as soon as possible and eventually fulfill the efficient implementation of theoretical research into clinical benefits. However, research at this stage is still highly geographically restricted, and participation in collaborative exchanges among international institutions is still low. At the same time, China lacks academic leaders who are as prolific and high-quality as Sessler Daniel I in the United States. We propose that collaboration in this field should transcend geographical and political barriers and Scholars should actively explore much more collaborative exchanges with their international counterparts. In recent years, with the deepening of the aging population and the pandemic of COVID-19, people's demand for surgery quality has increased more than ever, requiring not only that the surgery be completed, but also that it be done smoothly and safely, with faster recovery, reflecting the pursuit of comfort-oriented medical care. It is conceivable that the development of IOH research will be further accelerated in the near future as academic exchanges across regions and countries become closer. Among the top 10 productive journals, 9 of them were strongly associated with anesthesiology and for instance *Anesthesiology* and *Anesthesia and Analgesia* were both authoritative in the field of anesthesiology with a high quantity of publications and profound academic impact. The impact factor (IF) and quartile were obtained in JCR 2020 to assess the quality of journals in IOH field. Generally, most of the top cited journals were of exceptional quality, and 9 of them were in Q1 or Q2, and the rest of them in Q4.

### 4.2. Research hotspots and frontiers

Currently, there are many data suggesting a relationship between IOH and postoperative organ dysfunction and death (24, 25).Although no guidelines specify a risk threshold for IOH, a 2018 retrospective cohort study suggests that prolonged exposure (≥10 min) to MAP <80 mm Hg and for shorter durations <70 mm Hg was associated with mildly elevated risks of any end-organ injury. Other studies have further demonstrated that MAP below the absolute threshold of 65 mmHg or the relative threshold of 20% is associated with both myocardial and kidney damage (3). Based on these studies, *the 2019 Perioperative Quality Initiative Consensus Statement* concludes that during surgery anesthesiologists should maintain an MAP threshold greater than 60–70 mm Hg (26).

Arterial blood pressure is mainly affected by cardiac blood output and systemic vascular resistance, which is an extremely complex physiological variable usually described by systolic blood pressure (SBP), mean arterial pressure (MAP) and diastolic blood pressure (DBP). In order to better analyze the causes of IOH, in clinical practice, anaesthetists often use the model “BP = cardiac output (CO) *systemic vascular resistance (SVR), CO = heart rate (HR) *stroke volume (SV)”. Since the main factors influencing output per beat are myocardial contractility, venous return (preload) and arterial blood pressure (afterload), the variation in blood pressure can be considered as a multivariate function of heart rate, preload, afterload, myocardial contractility and systemic vascular resistance. Thus, several of the above factors can cause variation in blood pressure as well as lead to the development of IOH. The application of the model “BP = HR*SV*SVR” will help the researchers explore the causes of IOH more conveniently and adopt a more accurate clinical strategy and precise prediction of IOH.

The maintenance of proper blood pressure is crucial not only for ensuring adequate circulation and perfusion to vital organs such as the heart, brain, and kidneys but also for closely influencing the body's oxygenation status. As a controllable factor, the fluctuation and control of intraoperative blood pressure have received extensive attention. Recent studies have identified several major independent risk factors such as lower pre-induction SAP, older age, emergency surgery, supplementary administration of spinal or epidural anaesthetic techniques, male sex, and ASA PS IV (27–29). It has also been documented that chronic arterial hypertension and nocturnal non-dipping are highly associated with post-induction IOH in non-cardiac surgery patients (30). However, the existing prediction models are few, so it is urgent to develop a prediction model that comprehensively integrates each risk factor to provide clinical guidance.

A deep learning machine has been introduced to continuously monitor blood pressure during surgery, allowing for real-time warning before hypotension occurs (31). But other researchers have found that half of these alarms are not properly treated. Therefore, in 2021, Maheshwari et al. (32) further pointed out that the algorithm can only show better performance when using combined signals rather than single signals, and invasive monitoring is more reliable than non-invasive blood pressure monitoring (32). This also illustrates the need for more trials to enhance the clinical applicability of the algorithm, such as the addition of important surgical factors including comorbidities and surgical complexity (33), the use of lower alarm thresholds, simpler processing algorithms, and emphasis on timely treatment (32). A 2021 single-center prospective study reported that patients receiving goal-directed hemodynamic treatment regiments (fluid, vasophosphoric drugs, or positive inotropic drugs with an HPI of 85 or higher), as guided by the Hypotensive Predictive Index (HPI), had high blood pressure for a longer period of time, possibly as a result of overtreatment (34).

It has been reported that IOH often occurs during induction of anesthesia in hypertensive patients (35). Whether to stop hypertension drugs, such as ARB/ACEI, before operation is an important issue. The 2014 American Heart Association/American College of Cardiology Guidelines on perioperative cardiovascular Evaluation and Management in non-cardiac surgery patients state that continued use of ACE inhibitors during perioperative periods is justified, and if ACE inhibitors are retained prior to surgery, recommend that they be restarted postoperatively if clinically feasible (36). However, a 2018 systematic review confirmed that continued perioperative use of ARB/ACEI was associated with an increased incidence of IOH (37). A 2022 study found that regular use of ARB/ACEI with a long half-life increases the risk of hypotension during anesthesia induction in hypertensive patients, even after 24 h of withdrawal before surgery, so especially ARB/ACEI with a longer half-life than 24 h should be carefully adjusted by clinicians considering the pros and cons of withdrawal (38) and all perioperative risk factors (39). This was confirmed in a 2020 case of a patient who developed refractory hypotension during general anesthesia after withholding Telmisartan (40).

Various factors directly or indirectly influence the body's self-regulatory thresholds. Common clinical anesthetic drugs and vasoactive drugs have an important impact on blood flow regulation (8). Therefore, a preemptive increase in preoperative baseline values, such as by volumetric loading or induction with catecholamines, may mitigate the adverse hemodynamic effects after induction of anesthesia (27). A closed-loop vasopressor (CLV) controller was developed in 2019 to automatically adjust the norepinephrine infusion rate via continuously recorded MAP values in the arterial catheter, which is more effective in reducing IOH than intermittent push or manual adjustment of the infusion form (41). But there is still no consensus on which strategy or drug to use, and it depends more on the habits of the anesthesiologist.

IOH can be caused by many factors, such as vasodilation due to anesthetic drugs (42) or allergic reactions (43), intravascular hypovolemia due to intraoperative bleeding (44) or thrombosis (45), decreased cardiac output due to myocardial injury (46, 47), blood volume redistribution due to preganglionic sympathetic block (48), and catecholamine depletion from chronic cocaine use (49). It is also important to note that in 2019 Miller et al. found that severe intraoperative hypotension may also occur following the use of the Belmont Fluid Management System, a fluid management system for rapid infusion of blood products to the internal jugular vein (50).The cause still needs to be explored.

In the general population, intraoperative mean arterial pressure of less than 60–70 mmHg is associated with myocardial injury (51, 52), acute kidney injury (53, 54), and death (55) in adults. In contrast to specific blood pressure thresholds, there is evidence of a hierarchical effect between IOH and postoperative adverse outcomes (56), further confirming the clinical need to individualize the minimum acceptable blood pressure for patients or specific organs (57). The underlying etiological mechanism is still controversial. Wesselink et al. reported that the depth of hypotension contributed more to postoperative organ damage than the duration of IOH (56). However, consistent with previous studies, a retrospective observational study in 2023 indicated that hypotension duration > 5 min was associated with an increased risk of postoperative delirium in older adults (58). More randomized trials are needed to guide how to prevent and treat IOH.

At present, the mechanism of IOH is still diverse, and the consequences of improper treatment are unimaginable. Therefore, we should pay more attention strengthen the monitoring of patients during perioperative period, reduce the incidence of IOH, and adjust and maintain the intraoperative blood pressure in a rapid and appropriate way.

## 4.3. Limitation

We conduct statistical analysis of IOH trends, hot topics and cutting-edge issues over the past few years through three software: CiteSpace, VOSviewer, and R-bibliometrix. The discussion in this review still has many limitations. All the literature data here comes from the Web of Science only. While the Web of Science's up-to-date, authoritative, and extensive nature has provided us with a wealth of literature to support our analysis, we cannot rule out the possibility of missing a portion of the results on IOH, which other data engines cannot ignore. In addition, due to the copyright of the database, we can only collect the articles that have been included in WOSS database since 2004, which will inevitably avoid the neglect of excellent and authoritative literatures before 2004. Finally, this study is only for English documents, and non-English documents are not included in the collection criteria.

## 5. Conclusion

In this study, we searched for papers on intraoperative hypotension published in the Web of Science database from 2004 to 2022 and analyzed their basic information. Also, we conducted a relatively correlation analysis of the articles citations and furthermore chose the number of annual publications and citations, countries/regions, institutions, the productive and influential authors, the co-authorship and journals as reference indicators. In this respect, keywords and the gradual evolution of intraoperative hypotension research hotspots were evaluated.

Undoubtedly, the United States is regarded as the largest contributor and takes the principle position in global research. In the meanwhile, the collaborations of the Occident have been continuously enhanced, however, there is a lack of communication between the Asian countries and other international institutions. Among all authors, Sessler Daniel I takes the title of the most influential and prolific expert in IOH field, possessing with the highest publications and citations as well as willingness to collaborate with others. *Anesthesia and Analgesia* and *Anesthesiology* are the most significant journals in this field, accounting for a majority of size in publications and citations. Specifically, the mechanisms of IOH, the associations between IOH and any other complications such as AKI and MINS and the prevention and prediction of IOH will rule the results of these studies in IOH. To get a conclusion, our study shows a multi-dimensional bibliometric analysis of research on IOH from global perspective and may provide some useful clues for future directions of further research and scientific decision-making in this domain.

## Data Availability

The original contributions presented in the study are included in the article/Supplementary Material, further inquiries can be directed to the corresponding authors.
